# Incidence of respiratory syncytial virus infection in children with congenital heart disease undergoing immunoprophylaxis with palivizumab in Pará state, north region of Brazil

**DOI:** 10.1186/s12887-019-1681-6

**Published:** 2019-08-28

**Authors:** Roseane Porfírio de Souza, Andre Luis Ribeiro Ribeiro, Sílvio Augusto Fernandes de Menezes, Luiz Fernando Almeida Machado

**Affiliations:** 1 Biology of Infectious and Parasitic Agents Post-Graduate Program, Federal University of Pará, Belém, Pará Brazil; 2 Gaspar Vianna Clinic Hospital Foundation, Belém, Pará Brazil; 3 Postdoctoral fellowship, Cell Culture Laboratory, School of Dentistry, Federal University of Para – UFPA, Belém, Pará Brazil; 4 Clinical lecturer, Department of Periodontology, School of Dentistry, University Centre of Para – CESUPA, Belém, Pará Brazil; 5 Virology Laboratory, Institute of Biological Sciences, Federal University of Pará, Cidade Universitária Prof. José da Silveira Netto, Rua Augusto Correa 1, Guamá, 66.075-110, Belém, Pará Brazil

**Keywords:** Respiratory syncytial virus, Immunization, Palivizumab, Respiratory tract infections

## Abstract

**Background:**

Palivizumab prophylaxis for the human respiratory syncytial virus (HRSV) has been reported to reduce the risk of hospital admissions related to HRSV in children with congenital heart disease (CHD). These children are at high risk of developing severe lower respiratory tract infection (LRTI) due to HRSV infection. Our goal was to evaluate the incidence of HRSV infection in children with CHD after being submitted to immunoprophylaxis with palivizumab in Pará state, North region of Brazil.

**Methods:**

A prospective and observational cohort study was performed in children ≤2 years of age with CHD who received palivizumab immunoprophylaxis between January 1 and June 31, 2016. A questionnaire about basic non-medical care measures was applied to parents/legal representatives. Data on patients’ demographic characteristics, household environment, and respiratory infections were evaluated. HRSV infection was determined by qPCR.

**Results:**

There were 104 children enrolled in this investigation and the results showed a mean age of 10.6 months, an average weight of 7.3 kg and 3.5 doses of palivizumab per children during seasonality of HRSV. Respiratory infection was observed in 27.9% of cases, of which 9.6% were LRTI. No case of children who received palivizumab immunoprophylaxis and developed influenza-like symptoms tested positive for HRSV.

**Conclusion:**

Although the lack of a control group doesn’t allow to affirm the effectiveness of HRSV passive immunization, the immunoprophylaxis with palivizumab appeared to be totally efficient in preventing respiratory infection by HRSV in children up to two years of age with CHD.

## Background

The human respiratory syncytial virus (HRSV) is the most common etiologic agent of acute lower respiratory tract infection (LRTI) in neonates and children under five years of age worldwide [1] and it is the fourth cause of death in this group of children in Brazil [2]. HRSV infection is associated with risk factors such as overpopulation, pollution and malnutrition [3]. Children with congenital heart disease (CHD) are at high risk of developing acute LRTIs, and HRSV infection increases the chances of developing more severe LRTIs and extends the length of hospitalization because of respiratory complications. It increases not only the morbidity and mortality rates of these children but also the treatment costs due to higher admission rates in intensive care units, longer oxygen therapy and mechanical ventilation among those children [4]. It is estimated that 3.4 million hospitalizations and 199,000 deaths occur globally as a result of HRSV infection [1].

HRSV infections occur predominantly in well-defined seasons, usually during autumn and winter, and in temperate and subtropical regions. These seasons last between 16 to 20 weeks annually [5, 6] and often matches with the seasonality of the influenza virus [7]. The prevalence of HRSV infection in Brazil is derived from the data of the Influenza Sentinel Surveillance Information System and other respiratory viruses (SIVEP-FLIPE). It was based on data from influenza-like reports of illness during the period of 2007 to 2014. This data showed that HRSV infection has a different seasonality depending on the region in the country [8]. Based on that, the Ministry of Health (Joint Technical Note no. 05/2015) defined the regional seasonality of HRSV infection in different regions in Brazil, which in the North region corresponds to the period from January to June [8].

Although HRSV infection activates the immune system, acquired immunity does not prevent reinfection and vaccination with attenuated viruses appears to aggravate subsequent diseases [9]. Thus, the Ministry of Health of Brazil [10] has established measures to reduce the transmission of HRSV with passive immunization using the monoclonal antibody palivizumab (Synagis, MedImmune Laboratory, Gaithersburg, USA), which is composed of 95% human and 5% murine amino acid sequences. It was established by law and includes a population of preterm babies (< 28 weeks) who are not older than 12 months; and children up to two years of age with either CHD with hemodynamic significance or with chronic lung disease. Administration of palivizumab is performed intramuscularly at a dose of 15 mg/kg [11].

So far, no epidemiologic survey has been performed to check the efficacy of the palivizumab program since it’s beginning in Pará state. Thus, this study aimed to evaluate the incidence of HRSV in children (< 2 years of age) with CHD who were submitted to immunoprophylaxis with palivizumab in Belém, Pará, Brazil. This study also investigated the knowledge of children’s parents/legal representatives regarding basic non-medical care measures to reduce HRSV transmission; the efficiency of the palivizumab program and its recommended monthly doses; and analysed the incidence of respiratory infections caused by HRSV in children after palivizumab immunization in a specialized reference hospital in cardiology in Belém, Brazil.

## Materials and methods

### Study design, population studied and ethical aspects

This was a prospective observational cohort study involving children with CHD who were participating of the palivizumab program attending various sectors (e.g. outpatient clinic, neonatal intensive care unit, paediatric clinic and intensive care unit and paediatrics) of the Gaspar Vianna Public Foundation Hospital (FHCGV). This is specialized and a reference hospital in cardiology located in Belém, Pará, Brazil. The FHCGV carries out the assistance program for children with cardiopathy (PAPCC), where children receive outpatient care from nurses, social workers, psychologists, nutritionists, dentists, paediatricians and paediatric cardiologists. All children with CHD enrolled in the PAPCC whose parents/legal representatives agreed to join the study and received palivizumab immunoprophylaxis for HRSV from January to June 2016 were included in our sample.

This study was approved by the Ethics Committee of the Gaspar Vianna Public Foundation Hospital under protocol number 53017116.5.0000.0016, according to the resolution 466/2012 of the National Health Council. The parents or legal guardians of the participant children signed an informed consent form and answered questions regarding basic non-medical care measures to reduce HRSV transmission. The questionnaire included 8 qualitative yes / no questions.

### Inclusion and exclusion criteria

All children younger than 2 years of age from both sexes, who had CHD with confirmed haemodynamic significance and received at least one dose of palivizumab during the study period were included. To receive palivizumab immunoprophylaxis, the child should fill the criteria established by the FHCGV outpatient clinic: 1- to be selected according to the protocol for the palivizumab program; 2- to be prescribed by a paediatrician or paediatric cardiologist 3- to have determined the full treatment (number, timing and concentrations of palivizumab doses); 4- received the complete guidelines of treatment; 5- to have all palivizumab doses recorded on the medical records. Children who received the treatment but failed to return for taking subsequent doses or did not show up on follow-up appointments were excluded from our samples.

### Sampling and follow-up

On the day of administration of the first palivizumab dose, all children were weighted and reviewed by a paediatrician or paediatric cardiologist. Palivizumab doses were calculated and the overall clinical conditions checked before drug administration. Doses were calculated using the following formula: weight in kilos times 15 (ideal dose/kg of palivizumab) divided by 100 (the concentration of palivizumab in mg/mL in use). Hospitalized children were evaluated by the assistant paediatrician.

Palivizumab immunization was carried out in monthly doses and the total number of doses taken was based on the time of enrolment in the program. Children who were enrolled in January could take a total of 6 doses, however, those who enrolled after January were given the same monthly dose, which resulted in a lower number of total doses taken. Parents or legal representatives were invited to participate in this study and those who agreed to join in, signed the consent form and answered a questionnaire. An active search for clinical manifestations suggestive of HRSV infection post-immunoprophylaxis with palivizumab was carried out through phone calls monthly.

### Palivizumab treatment records

A structured form was used to record the palivizumab doses taken and post-immunoprophylaxis follow-up. During the study, it was established that any child who developed influenza-like symptoms (cough or sore throat, rhinorrhoea, coryza and malaise) should come to the outpatient clinic of the FHCGV from Monday to Friday, no later than the fifth day of the beginning of symptoms. All children with influenza-like symptoms were tested according to the protocol of the Global Influenza Program [12] to investigate respiratory infection caused by HRSV, which also included those children who developed LRTI and required hospitalization. Tests for HRSV infection and other respiratory viruses were performed in the Central Public Health Laboratory of Pará (LACEN-PA).

HRSV infection was determined by quantitative polymerase chain reaction (qPCR), which is considered one of the most accurate tests for detecting HSRV strains [13]. Amplicons signals were measured relative to the internal reference dye (ROX) to normalize for non-PCR-related fluorescence fluctuations occurring from well to well. The qPCR protocol used was standardized by the Centre for Disease Control and Prevention (CDC), Ministry of Health of Brazil and its efficiency has been largely tested across the country. Positive results were determined by the fluorescence intensity values of our samples compared to a positive control previously established by the CDC.

Data from cases that developed LRTI caused by HRSV were retrieved from medical records. Information regarding the length of stay during hospitalization, number of doses of palivizumab received, use of supplementary oxygen and mechanical ventilation, intensive care unit (ICU) admission, and death was collected. Furthermore, additional information included the seasonality of the HSRV infection, the beginning and ending of treatment, and other upper and lower respiratory tract infection. In this study, the cities of Ananindeua, Marituba, Benevides, Santa Bárbara do Pará, Santa Isabel do Pará and Castanhal were considered as the Belém metropolitan area.

### Data analysis

Data were inputted on Microsoft® Access 2010 database and summarized in tables. The following variables were considered: the geographical location, other preventive HSRV infection measures (apart from palivizumab immunoprophylaxis), children’s demographic data on the day of the first dose (age, gender, weight), follow-up period, volume and number of doses of palivizumab prescribed.

## Results

There were 104 children who fulfilled the inclusion criteria and received immunoprophylaxis with palivizumab from January to June 2016 at the FHCGV in Belém, Pará. One child was excluded because the treatment with palivizumab was administered for a non-cardiologic reason.

Eight questions were asked to children’s parents or legal representatives about their knowledge of basic non-medical measures to reduce the transmissibility of HRSV. Results showed that 77% (80/104) of the children’s parents or legal representatives knew about the need of washing hands to prevent virus transmission; 70% (73/104) performed hands hygiene before and after contact with the child; and 69% (72/104) had been advised of avoiding exposure of the child to passive smoking. Answers to all questions of the questionnaire are in Table 1.Two-thirds of these measures were known by children’s parents or legal representatives and less than half of them were aware of vaccination against influenza virus as preventive care.

**Table 1:** Analysis of the knowledge of parents and legal representatives of children who received immunoprophylaxis with palivizumab at the FHCGV from January to June of 2016 on general measures of non-drug basic care to reduce the transmissibility of HRSV

Preventive HRSV infection measures	n	%
Appropriated hand hygiene	80	76.9
Cleaning of hands before and after contact with the child	73	70.2
Avoid exposure of the child to passive smoking	72	69.2
Reinforcement of the child personal hygiene	71	68.3
Disinfection of contaminated surfaces	71	68.3
Limit contact with people with a respiratory infection	69	66.4
Avoid agglomerations	67	64.4
Vaccination against influenza virus	49	47.1
Total		66.3

There were 58% (60/104) of males and 42% (44/104) of females. The geographical location indicated that 54% (56/104) of participants came from the countryside, 29% (30/104) lived in the city of Belém, and 17% (18/104) were residents from the Belém metropolitan area. The FHCGV outpatient clinic was responsible for 81% (84/104) of the source of participants, 10% (11/104) was assisted on the paediatric clinic, 6% (6/104) came from neonatal ICU and 3% (3/104) from other sectors of the hospital. The beginning of immunoprophylaxis with palivizumab was in January for 32% (33/104) of the cases, followed by February with 26% (27/104) and March with 19% (20/104).

Palivizumab immunoprophylaxis was given only during the regional epidemic season of HRSV (January to June). Children who started the immunization in January had a chance to take all the six doses; the others were given monthly doses according to the month of enrolment until June. Of all participating children, only 17 (16.3%) had received 6 doses of palivizumab during the study and 24 (23.0%) received two doses. Demographic data including palivizumab treatment information is shown in Table 2.

**Table 2:** Demographic data of children with CHD who received immunoprophylaxis with palivizumab at the FHCGV from January to June 2016, in Belém, Pará, Brazil

Characteristics	n	%
Gender		
Male	60	57.7
Female	44	42.3
Geographic origin		
Belém	30	28.8
Belém metropolitan area	18	17.3
Countryside	56	53.9
Hospital sector of origin		
Outpatient clinics	84	80.8
Esternal outpatient clinics	3	2.9
Paeditrics clinics	11	10.5
Neonate ICU	6	5.8
Month of the beginning of immunoprophylaxis		
January	33	31.7
February	27	26.0
March	20	19.2
April	10	9.6
May	10	9.6
June	4	3.8
Number of palivizumab doses taken		
1 dose	16	15.4
2 doses	24	23.1
3 doses	15	14.4
4 doses	13	12.5
5 doses	19	18.3
6 doses	17	16.3

The average age during the beginning of palivizumab immunoprophylaxis was 10.6 months (SD ± 6.6), mean weight was 7.3 kg (SD ± 2.7), average dose of palivizumab was of 1.1 mL (SD ± 0.4) and the mean number of doses given during the study was 3.5 (SD ± 1.7).

Medical records showed that 28% (29/104) of children had at least one episode of respiratory tract infection, with a ratio of 1.3 episodes of infection per patient (SD ± 0.4). It resulted in 40 respiratory infections during the period of study.

Thirty out of 40 respiratory infections (75%) were on the upper respiratory tract and 10 (25%) involved the lower respiratory tract (Table 3). The mean length of stay in the hospital due to these infections was 51 days (SD ± 35). From those children who developed LRTI, 10% (1/10) presented bronchiolitis, 50% (5/10) had pneumonia, and 10% (1/10) developed both, bronchiolitis and pneumonia. Admission to ICU due to LRTI was required for 50% (5/10) of children and the average length of stay in the ICU was 24 days. From these children, 40% (4/10) required only oxygen therapy and 30% (3/10) needed mechanical ventilation (Table 4). During the LRTI, the nasopharyngeal aspirate was positive for metapneumovirus in 20% (2/10) of the cases. In the follow-up period, one child has passed away 0.9% (1/104) due to a non-respiratory cause after cardiac surgery.

**Table 3:** Incidence of respiratory tract infection in 104 children with congenital heart disease after immunoprophylaxis with palivizumab in Belém, Brazil from January to June 2016

Respiratory infection information	n	%
Upper respiratory tract infection (URTI)	30	28.8
Lower respiratory tract infection (LRTI)	10	9.6
Diagnosis in outpatient clinic	27	26.0
LRTI acquired during hospitalization	3	2.9
Hospitalization due to respiratory infection	7	6.8

**Table 4:** Diagnostic and medical care needed for treating 10 children who developed lower respiratory tract infection (LRTI) after immunoprophylaxis with palivizumab in the Belém, Brazil from January to June 2016

LRTI information	n	%
Pneumonia	5	50
Metapneumovirus infection	2	20
Bronchiolitis	1	10
Bronchiolitis and pneumonia	1	10
ICU admission	5	50
Oxygen therapy use	4	40
Mechanical ventilation use	3	30

## Discussion

To our knowledge, this is the first study in the North region of Brazil that investigated the incidence of HRSV infection after the use of palivizumab passive immunization in children with CHD. Our results showed that there was not even a single case of HRSV positive test after palivizumab immunization.

Although the incidence of HRSV in children with CHD in this region is not known, data from the influenza sentinel surveillance system (SIVEP-Gripe) indicated a prevalence of 9.6% of HSRV positivity in children younger than 5 years of age with influenza-like symptoms [14]. Other studies that focused on a population with a similar age (younger than 2 years), showed a prevalence of 52% of HRSV infection in children with acute respiratory tract infections (São Paulo, Brazil) [15] and 40.2% in children with LRTI in the Northeast region [16]. Since 29 (27.9%) of the participant children in this study had a respiratory infection, it was expected that at least a few children would be infected by the HSRV, the most common aetiological agent in this type of infections [15, 16]. However, no case of HRSV infection was identified in our sample, suggesting that palivizumab immunization was effective in preventing respiratory infections caused by HRSV.

Children started immunoprophylaxis with palivizumab with a mean age of 10.6 months, which was older than that found in São Paulo (8.4 months) [17] and in children with heart disease in Korea (2.9 months) [18]. The mean weight of the participant children during the first dose of treatment was 7.3 kg and resulted in an average dose of 1.1 mL of palivizumab (100 mg/mL), in accordance with the standards of the Brazilian Ministry of Health [10].

The average number of doses of palivizumab injections during the study period was 3.5 doses/child, similar to that reported in Korea (3.7 doses) [18], Latin America (3.8 doses) [19] and in São Paulo (4 doses) [17]. Five doses of palivizumab were given to 34.6% of children, a percentage higher than reported in São Paulo (22.7%) [17] but lower than in Latin America (43.7%) [19]. Since palivizumab is only administered during the HRSV epidemic season (January to June in the North region of Brazil), the reduced number of doses could be a result of the difficulties of countryside children travelling to Belém in order to take their medication. It is important to mention that the Pará state has a huge territorial extension and the transportation system is poor, making the commuting from their hometown to Belém a challenging task. Thus, only children who started immunoprophylaxis during the months of January and February had the chance of receiving 5 doses.

There were 40 respiratory infections in 29 children (28%) with predominantly upper respiratory tract infections. Similar results were observed in Argentina (32%) [20] and much lower than in Korean children (61%) [18], Italy (76.4%) [21] and São Paulo (59.1%) [17]. We also identified an overall low incidence of LRTI (9.6%), which is much lower than that reported in São Paulo (40.6%) [17]. However, it may reflect differences in the studied population that did not include children with chronic lung disease in our study. Hospitalization was barely required (6.8%), which was lower than São Paulo (9%) [17], and even much lower than in Italy (22.9%) [21], Argentina (31%) [20] and Korea (60%) [18].

All children participating in this study had taken at least 1 dose palivizumab immunoprophylaxis while free of respiratory infections. Those children who developed a respiratory infection were previously passively immunized against HRSV. Since the immunization is passive, maintenance of the regular monthly immunization is the key point for preventing HRSV infection and it is not related to the total number of doses.

Although uncommon, when hospitalization was required, patients stayed for a long period (average of 51 days). It reflects the vulnerability of this specific population that have other medical problems requiring treatment, which can be aggravated due to their susceptibility to several other viral and bacterial infections. This long hospitalization stay is a result of a combination of other associated medical problems and excessive bureaucracy and slowness of the Brazilian public health system.

The respiratory infections found in this study were classified as bronchiolitis in 10% of cases, lower than in São Paulo (50%) [17] and Italy (22.9%) [21]. We did not observe any case of HRSV infection, suggesting a full efficacy of palivizumab immunoprophylaxis. However, we can not affirm its effectiveness since we did not include a control group of similar patients who did not take the passive immunization with palivizumab.

## Conclusion

The use of palivizumab immunoprophylaxis appeared to be effective in preventing HRSV infection, especially the LRTI in children CHD. Although respiratory infections were present in 27.9% of children enrolled in our study, none of them tested positive to HRSV, which reinforces the suggested efficacy of the palivizumab passive immunization, carried out by the Brazilian Ministry of Health for children with CHD, in preventing the incidence and complications related to respiratory infections caused by HRSV.

## Data Availability

Not applicable.

## Abbreviations

CHD: congenital heart disease
FHCGV: Gaspar Vianna Public Foundation Hospital
HRSV: human respiratory syncytial virus
LACEN-PA: Central Public Health Laboratory of Pará; ICU: intensive care unit
LRTI: lower respiratory tract infections
PAPCC: assistance program for children with cardiopathy
SIVEP-FLIPE: Influenza Sentinel Surveillance Information System and other respiratory viruses
